# P-2275. Impact of Epstein-Barr Virus (EBV) Donor Serostatus on Post-transplant Mortality and Post-Transplant Lymphoproliferative Disorder in Thoracic Organ Transplant EBV-Seropositive Recipients: Data from the Organ Procurement and Transplantation Network

**DOI:** 10.1093/ofid/ofae631.2428

**Published:** 2025-01-29

**Authors:** Khuloud Aldhaheri, Allison Mah, Alissa J Inc, Stephen Lee, Sara Belga

**Affiliations:** University of British Columbia; University of British Columbia; University of British Columbia; University of Saskatchewan, Regina, Saskatchewan, Canada; University of British Columbia

## Abstract

**Background:**

Epstein Barr Virus (EBV) donor positive (D+) serostatus is a risk factor for Post-Transplant Lymphoproliferative Disorder (PTLD) in EBV-seronegative recipients. The impact of donor EBV serostatus on mortality and PTLD in EBV-seropositive recipients (R+) in thoracic organ transplant (TOT) is unknown.

**Table 1.**
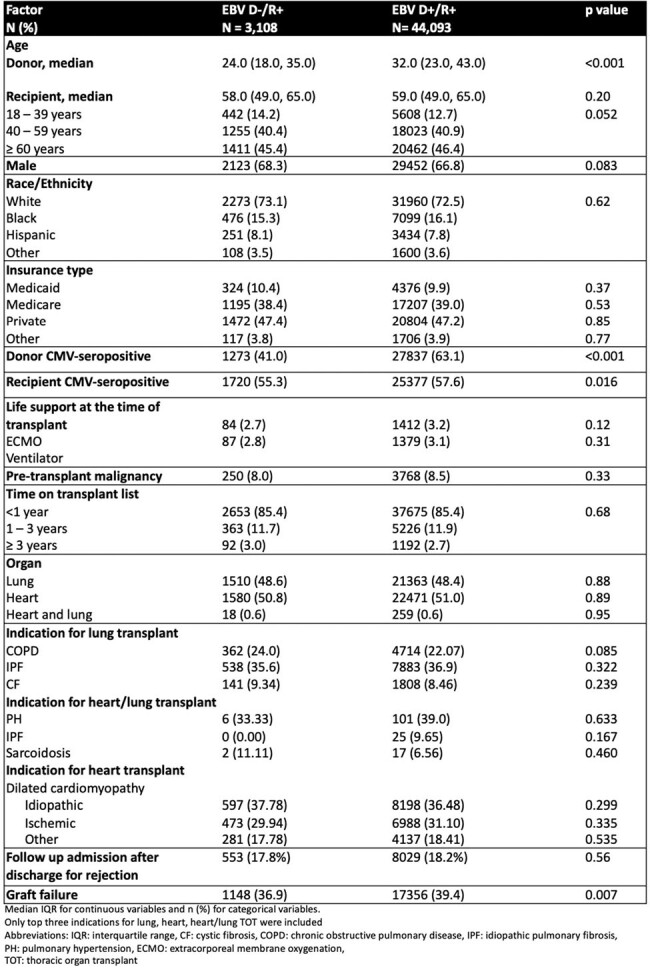
Baseline demographic and clinical characteristics of EBV D-/R+ and EBV D+/R+ TOT recipients

**Methods:**

Using the Organ Procurement and Transplantation Network database, we identified 47,201 EBV R+ TOT recipients between 01/2004 – 12/2021. The primary exposure was EBV D+ and the primary outcomes were death and PTLD. Multivariable Cox regression models were used to assess the relationship between donor EBV serostatus and the outcome of death and PTLD.

**Figure 1.**
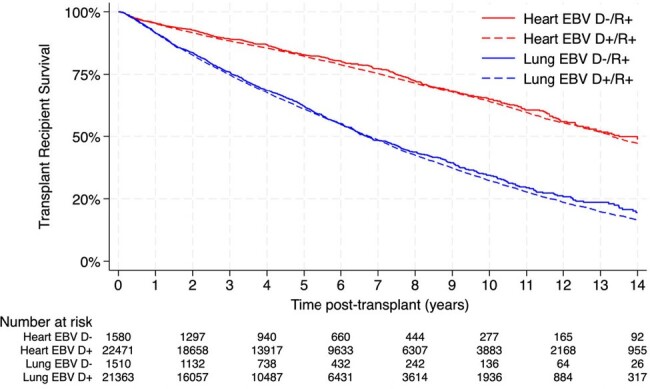
Kaplan-Meier survival plots for EBV D-/R+ (solid line) versus EBV D+/R+ (dashed line) post heart (red line) and lung (blue line) transplantation

**Results:**

Of 47,201 EBV R+ TOT recipients, 48.5% were lung and 51% were heart transplant recipients. The majority (93.4%) were EBV D+/R+, with median age of 59 years and 66.8% males. EBV D-/R+ and D+/R+ TOT recipients differed in CMV donor and recipient status, donor median age, sex, and graft failure (Table 1). The incidence rate of death was 7.2 per 100 person-years in EBV D+/R+ compared to 6.9 per 100 person-years in EBV D-/R+, *p*=0.156. Survival did not differ by EBV donor status, but it was lower in lung transplant (Figure 1). The incidence rate of PTLD was 0.26 per 100-person years in EBV D+/R+ compared to 0.33 per 100-person years in EBV D-/R+, *p*= 0.073. EBV D+ status was not associated with increased hazard of death in both univariable (crude hazard ratio [HR] of 1.05; confidence interval [CI], 0.98-1.11; *p*=0.15) and multivariable analyses (adjusted HR of 0.96; 95% CI, 0.90-1.02; *p*=0.14), after controlling for donor age, CMV D+ status, and the following recipient factors: age, sex, race, insurance, pre-transplant life support, pre-transplant malignancy, rejection, and graft failure. EBV D+ status was associated with decreased hazard of PTLD when adjusting for recipient age and graft failure (adjusted HR of 0.74; 95% CI, 0.56-0.98; *p*=0.03). In subgroup analyses by organ, EBV D+ status was associated with decreased hazard of PTLD in lung (adjusted HR of 0.56; 95% CI, 0.40-0.78; *p*=0.001) but not in heart transplant recipients (adjusted HR of 1.18; 95% CI, 0.71-1.96; *p* =0.50) (Figure 2).

**Figure 2.**
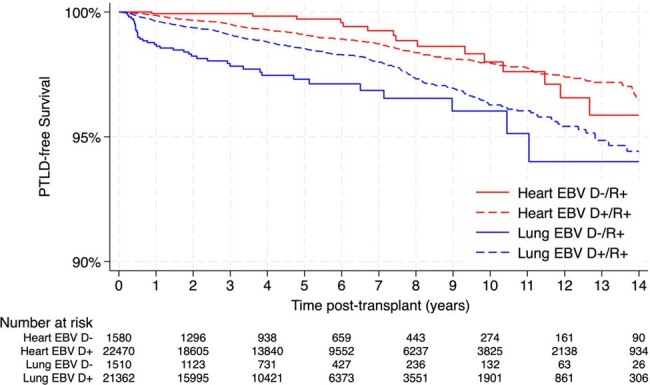
Kaplan-Meier PTLD-free survival plots for EBV D-/R+ (solid line) versus EBV D+/R+ (dashed line) post heart (red line) and lung (blue line) transplantation

**Conclusion:**

Among EBV-seropositive TOT recipients, EBV D- status may be associated with increased risk of PTLD, particularly in lung transplant recipients.

**Disclosures:**

Alissa J. Inc, MD, MSc, Takeda: Honoraria Sara Belga, MD, MPH, Takeda, Moderna, AstraZeneca, GSK: Advisor/Consultant|Takeda, Moderna, AstraZeneca, GSK: Grant/Research Support|Takeda, Moderna, AstraZeneca, GSK: Honoraria

